# Speckle tracking echocardiographic prediction of atrial fibrillation after patent foramen ovale closure: a challenging matter

**DOI:** 10.1093/ehjimp/qyae032

**Published:** 2024-04-25

**Authors:** Antonio Vitarelli, Lidia Capotosto, Gaetano Tanzilli, Enrico Mangieri

**Affiliations:** Department of Echocardiography, Cardiodiagnostica CS, Via Lima 35, 00198 Rome, Italy; Department of Cardiology and Cardiac Surgery, Sapienza University, v.le Policlinico 155, 00185 Rome, Italy; Department of Echocardiography, Cardiodiagnostica CS, Via Lima 35, 00198 Rome, Italy; Department of Cardiology and Cardiac Surgery, Sapienza University, v.le Policlinico 155, 00185 Rome, Italy; Department of Cardiology and Cardiac Surgery, Sapienza University, v.le Policlinico 155, 00185 Rome, Italy; Department of Cardiology and Cardiac Surgery, Sapienza University, v.le Policlinico 155, 00185 Rome, Italy

There is growing evidence that transcatheter percutaneous closure of patent foramen ovale (PFO) plus medical therapy should be performed in patients with cryptogenic stroke or recurrent transient ischaemic attack rather than anticoagulant or antiplatelet therapy alone. Therefore, we read the paper by Tarsia *et al.*^1^ with great interest. Given the recent evolution of ultrasound equipment and processing tools, the purpose of comparing biatrial function assessed by speckle tracking echocardiography (STE) after PFO closure by direct suture vs. closure with traditional occluders was brilliant and the authors are to be congratulated for conducting this prospective study. However, we believe it is worthwhile to clarify or explore further some issues regarding previous literature and the article itself.

In the paper^2^ cited by the authors, we investigated atrial mechanics in patients with PFO and atrial septal defect (ASD) before and after occluder placement. We found that in patients who underwent successful device closure, pre-existing atrial dilation and dyssynchrony had a higher association with paroxysmal atrial fibrillation (PAF) than the size of the implanted device. The overall incidence of PAF after 6 months of follow-up in ASD device patients (24%) was higher than the PFO device group (13%). The pathogenesis of atrial fibrillation (AF) is complex and multifactorial, and potential mechanisms include not only local stretching or irritation resulting from the device itself but also intrinsic patient factors such as pre-existing atrial enlargement or a chronic biatrial substrate that may be necessary to sustain even relatively short episodes of AF. Combined right atrial (RA) stretch and ventricular overload presumably explain vulnerability to atrial tachyarrhythmias in ASD patients. Some degree of left atrial (LA) dysfunction, such as impairment of active or passive emptying and contraction/reservoir functions,^2–4^ was described in patients with PFO, especially those with moderate to large atrial septal aneurysm (ASA). We have shown distinctive predictive RA vs. LA values to differentiate ASD from PFO in terms of PAF occurrence.^2^

Further considerations suggest that pre-closure atrial changes are implicated in the development of PAF after atrial septal procedures in addition to device size and that the short- and long-term risk of developing AF in patients with device implantation is an intricate issue. It was shown that in patients with cryptogenic stroke, the benefit of closing a PFO is related to the morphological complexity of high-risk PFO assessed by transoesophageal echocardiography,^5^ and some meta-analysis studies reported that PFO closure may be associated with reduction in the prevalence of AF. In patients older than 60 years with comorbidities, PFO device implantation was associated with a lower incidence of recurrent ischaemic events compared with patients who did not undergo PFO closure, although with higher rates of recurrent events and new-onset AF than younger patients.^6^ It was also shown that patients with PFO device had an increased risk of developing AF within the first 3 months but not thereafter up to 5 years.^7^ Thus, the clinical significance of new-onset ‘secondary’ AF in PFO patients remains an open question.

In the paper by Tarsia *et al.*,^1^ patients who underwent occluder implantation had significantly worst strain indices and different minimal volumes compared with baseline at 1-year follow-up, whereas in patients with suture-mediated PFO closure, no differences were observed in the same parameters. We should be grateful for their contributions to an understudied topic, even if some concerns persist. It can be hypothesized that these findings were related to the rigorous patient selection that led to the exclusion of patients with ASA and reduced possible causes of LA dysfunction pre-existing the device plant. Furthermore, strain analysis was not performed by sector and, as the authors acknowledge, functional data limited to the septum were not collected in isolation in patients with occluder device. Although a recent consensus paper of the European Association of Cardiovascular Imaging/American Society of Echocardiography (EACVI/ASE) recommends the use of total rather than segmental wall strain to assess thin atrial wall function, segmental evaluation may possibly be preferable when looking for specific regional deformation. No adverse outcomes were reported except for higher residual right-to-left shunt in the HeartStitch group (16%) compared with the occluder group (8%) and asymptomatic periods of AF in one patient in the occluder group at 1–3 months after the procedure. Therefore, it was suggested to perform Holter monitoring twice a year after the occluder implantation, also in asymptomatic patients.

We suggest that, in addition to ECG monitoring and clinical risk factors (included in CHA2DS2-VASc-score) as AF predictors, STE can help detect LA structural and functional changes in patients with potentially underlying arrhythmia (*Figure 1*). In our experience, peak strain, time-to-peak strain, and expansion index were the most sensitive indices for PAF prediction (*Table 1*). Left atrial reservoir strain was assessed with zero strain reference set at left ventricular end-diastole (R-R gating). Complementary assessment of atrial strain/dyssynchrony and volumetric parameters represented the most accurate screening strategy to predict the development of AF. Three-dimensional (3D) volumetry predicted PAF in the presence of atrial enlargement better than two-dimensional (2D) biplane indices obtained by LA-focused views; however, 2D methods produced acceptable results and may also give useful information for identifying patients with potential PAF in the absence of 3D echocardiography. More research will come. Since structural heart disease intervention is an evolving field both for implementation of new devices and application of new sophisticated imaging techniques, we stress the importance of cooperation between the echocardiologist and interventional cardiologist and, in selected cases, the cardiac electrophysiologist for a comprehensive diagnostic and therapeutic/interventional approach to these patients.

**Figure 1:**
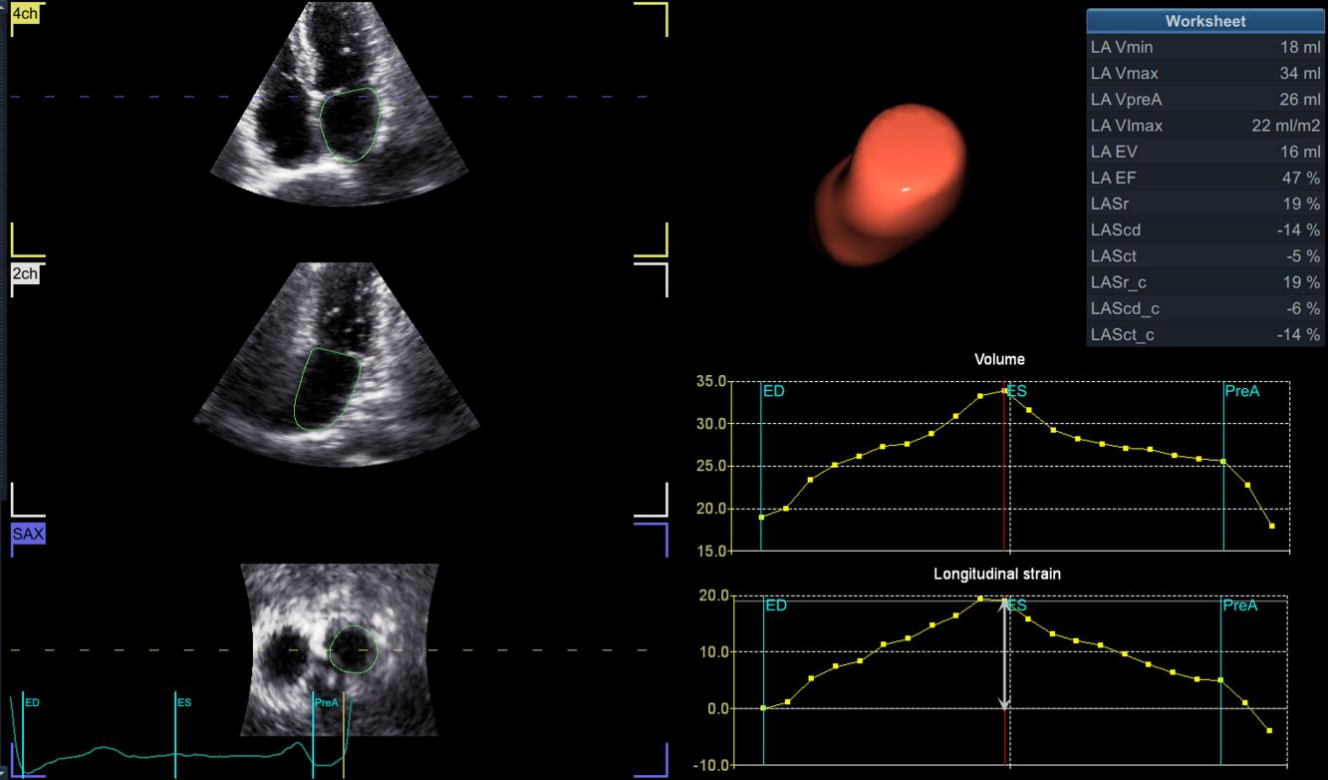
Three-dimensional speckle tracking echocardiographic quantification of left atrial volumes and function. (Left) Longitudinal and transverse visualization of three-dimensional echocardiographic left atrial volume. (Right) Left atrial volume–time and strain–time curves showing changes during the cardiac cycle. LA, left atrium; LAEF, total emptying fraction (total EV/Vmax); LAEV, total emptying volume (Vmax − Vmin); LAScd, longitudinal strain conduit phase; LAScd_c, circumferential strain conduit phase; LASct, longitudinal strain contraction phase; LASct_c, circumferential strain contraction phase; LASr, longitudinal strain reservoir phase; LASr_c, circumferential strain reservoir phase; LA-VImax, maximum volume index; LA-Vmax, maximum volume (at LV end-systole); LA-Vmin, minimum volume (at LV end-diastole); LA-VpreA, volume before atrial contraction (at LV early diastole). LA expansion index, LAEV/Vmin × 100.

**Table 1:** Results of receiver operating characteristic curves comparing device size and pre-closure echocardiographic parameters for their accuracy to predict PAF

Variable	AUC	95% CI	*P* value	Cut-off	Sensitivity	Specificity
Device size, mm	0.592	0.546–0.781	0.071	27	53	56
2D-LA-Vmax, mL/m^2^	0.646	0.604–0.782	0.053	33	58	83
2D-LA-Vmin, mL/m^2^	0.752	0.665–0.811	0.046	13	75	78
2D-LA-EI, %	0.794	0.687–0.842	0.043	107	79	77
LA-PS, %	0.831	0.692–0.884	0.045	25	82	76
LA-TPS, ms	0.861	0.748–0.898	0.024	119	88	85
3D-LA-Vmax, mL/m^2^	0.785	0.705–0.832	0.047	35	62	85
3D-LA-Vmin, mL/m^2^	0.834	0.726–0.885	0.031	15	82	80
3D-LA-EI, %	0.854	0.712–0.897	0.023	121	87	84
3D-LA-EI + LA-TPS	0.914	0.811–0.939	0.011	121 119	91	86

Adapted from Vitarelli *et al.*^2^

3D, three-dimensional; 2D, two-dimensional; AUC, area under the curve; CI, confidence interval; EI, expansion index; LA, left atrium; PAF, paroxysmal atrial fibrillation; PS, peak strain (reservoir phase); TPS, time-to-peak strain; Vmax, maximal volume index; Vmin, minimal volume index.

## Lead author biography


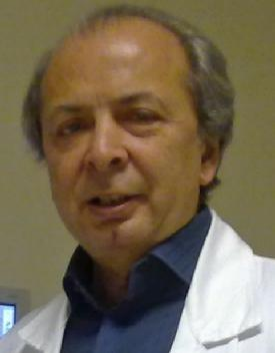


Antonio Vitarelli is an echocardiologist in Rome. He has been working at Toronto, Lund, Charlottesville, Detroit, and Chicago Universities. He was Director of the Echocardiology Operative Unit (clinical and research unit) from 1992 to 2013 at the Sapienza University Hospital of Rome and is currently consultant cardiologist and adjunct professor. He is a fellow of the American College of Cardiology and a senior member of various cardiac societies, has written more than 200 articles and a textbook on colour Doppler echocardiography, and is a reviewer for several international journals. His main scientific interests include advanced echocardiography, adult congenital heart disease, valvular heart disease, and cardiac function.

## Data Availability

The data underlying this article cannot be shared publicly due to ethical restrictions. Anonymized data are available to researchers on reasonable request to the corresponding author.
